# Gastric Mucormycosis in a Renal Transplant Patient Treated with Isavuconazole Monotherapy

**DOI:** 10.1155/2019/9839780

**Published:** 2019-03-17

**Authors:** Imran Gani, Atbin Doroodchi, Kristina Falkenstrom, Holly Berry, Won Lee, Laura Mulloy, Muhammad Saeed, Rajan Kapoor

**Affiliations:** ^1^Department of Nephrology, Hypertension and Transplant Medicine, Augusta University Health, Augusta, GA, USA; ^2^Division of Transplant Surgery, Department of Surgery, Augusta University Health, Augusta, GA, USA; ^3^Department of Pharmacy, Augusta University Health, Augusta, GA, USA; ^4^Department of Pathology, Augusta University Health, Augusta, GA, USA

## Abstract

Gastrointestinal mucormycosis is a rare infection in solid organ transplant recipients. Our patient, a 79-year-old male, presented with severe dysphagia and odynophagia about 2 weeks after receiving a renal transplant. An upper gastrointestinal (UGI) endoscopy revealed esophagitis and gastric ulceration, the cultures from which grew Rhizopus species. A usual treatment strategy should include Amphotericin B as monotherapy or in combination with Posaconazole or Isavuconazole for such infections. Our patient was treated with Isavuconazole monotherapy, in an effort to minimize renal toxicity from Amphotericin B to the new allograft. Unique to our case was a successful clinical response and resolution of UGI lesions with Isavuconazole monotherapy. Due to the vagueness of presenting symptoms, such infections can be easily missed in an immunocompromised patient which can have tragic outcomes. Prompt diagnosis and modulation of immunosuppression are essential to decrease mortality and morbidity. Isavuconazole is a novel agent and can be used as a monotherapy for such infections, especially in renal transplant recipients.

## 1. Introduction

Mucormycosis has emerged as a debilitating infection in renal transplant patients with a high incidence of allograft loss if the infection is disseminated. It carries high morbidity and mortality rates despite treatment. Rhizopus species has been reported to be the most common cause of mucormycosis infection in immunocompromised patients. Isavuconazole so far has not been used as monotherapy for the first line treatment for gastric mucormycosis in a renal transplant patient due to lack of clinical data.

We present a rare case scenario of a 79-year-old African-American male who developed severe gastrointestinal mucormycosis from Rhizopus species infection 2 weeks after receiving a renal transplant and was successfully treated with Isavuconazole monotherapy.

## 2. Case Report

Our patient, a 79-year-old African-American male with a past medical history of end-stage renal disease secondary to hypertension, DM Type 2, coronary artery disease received an uneventful deceased donor kidney transplantation. His induction immunosuppression consisted of antithymocyte immunoglobulin and steroids and his maintenance regimen consisted of Mycophenolate Mofetil, Tacrolimus, and Prednisone. He received Trimethoprim-Sulfamethoxazole, Valgancyclovir, and Nystatin for opportunistic infection prophylaxis. His immediate posttransplant course was complicated by transient delayed graft function and Clostridium difficile diarrhea which resolved after treatment by postoperative day 10.

**Figure 1:**
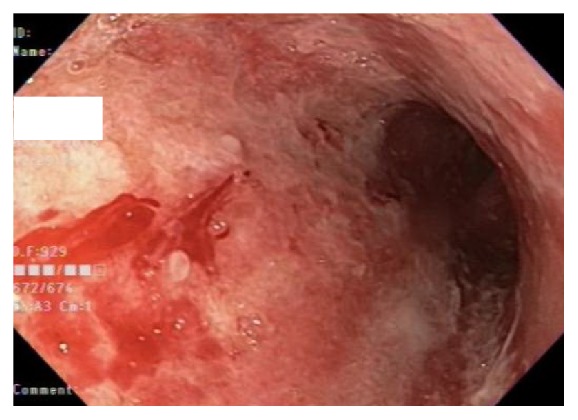
Endoscopic imaging showing severe esophagitis.

**Figure 2:**
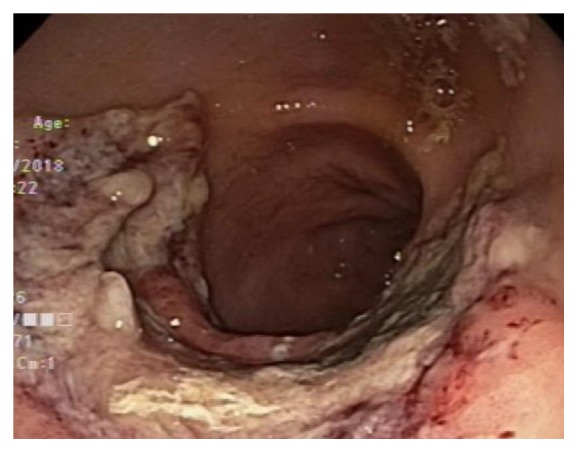
Endoscopic imaging of gastric lesion before the initiation of treatment.

**Figure 3:**
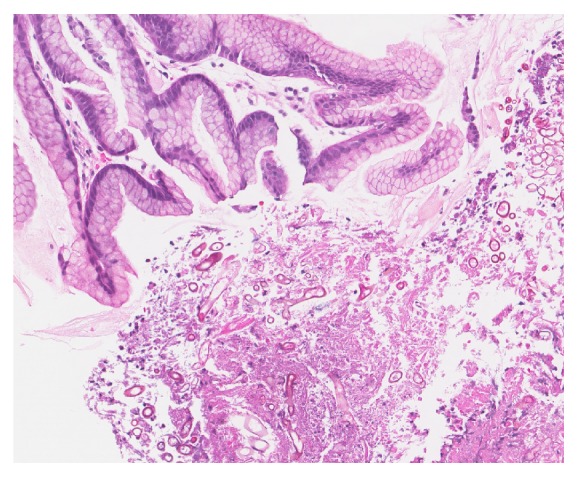
Fungal elements in a background of necrotic and acute inflammatory exudate and unremarkable gastric foveolar epithelia. No evidence of malignancy (x2000).

**Figure 4:**
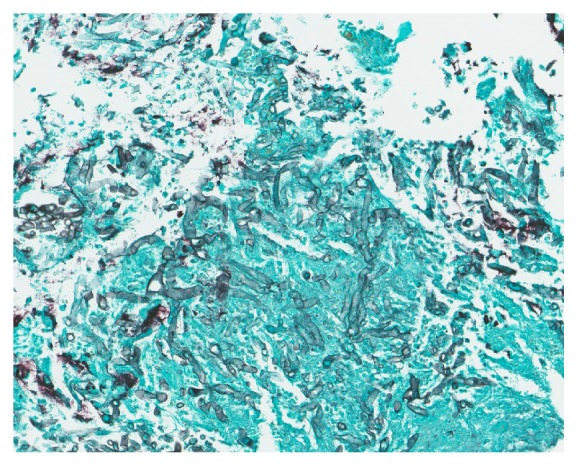
Fungal hyphae highlighted by Grocott-Gomori's Methenamine Silver (GMS) stain in a background of necrotic and acute inflammatory exudate and unremarkable gastric foveolar epithelia. No evidence of malignancy (x1000).

**Figure 5:**
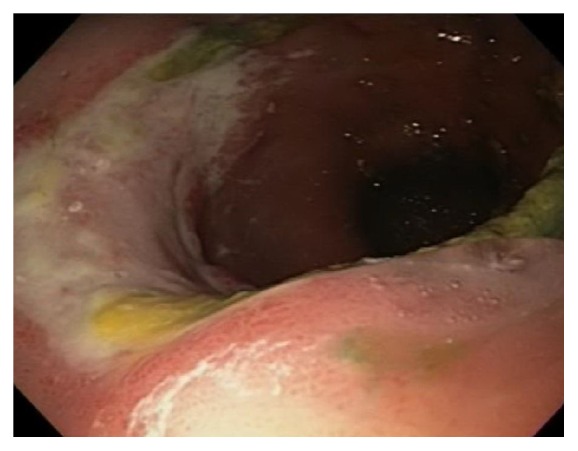
Endoscopic images of the gastric lesion after treatment with Isavuconazole.

On postoperative day 16, he started experiencing dysphagia and odynophagia and was unable to take solid food. An esophagogastroduodenoscopy (EGD) was performed revealing Los Angeles Grade D esophagitis, 20 cm in length (Figure 1), along with a large semicircumferential gastric ulcer with heaped up margins covered by greenish exudate (Figure 2). Histologic examination of the biopsy specimen revealed fungal elements in the background of necrotic and acute inflammatory exudate with unremarkable gastric foveolar epithelium (Figures 3 and 4). CMV and HSV stains were negative and the biopsy was negative for H. pylori and malignancy as well. Fungal culture grew Rhizopus species. The patient was started on Isavuconazole (372 mg every eight hours for 6 doses followed by 372 mg daily) and the dose of Mycophenolate Mofetil and Tacrolimus was reduced. He started experiencing resolution of symptoms in 48 hours and was able to tolerate oral feeds well. A repeat EGD on postoperative day 20 showed partial resolution of the mass (Figure 5). The patient was put on lifelong Isavuconazole (372 mg p.o daily) given the patient's immunosuppressed status and he has remained asymptomatic at 6 months after transplant, which was his last clinic follow-up visit.

## 3. Discussion

Gastrointestinal (GI) infections are common in recipients of solid organ transplant patients due to underlying immunosuppression. Common causes include Clostridium difficile, Cytomegalovirus, Herpes Simplex Virus, Helicobacter pylori, and enteric bacteria (Campylobacter, Escherichia coli, Salmonella). Other infectious agents implicated include parasites (Giardia intestinalis, Strongyloidiasis) and viruses (Norovirus and Rotavirus) [1, 2]. The most common cause of gastrointestinal fungal infection in transplant patients is Candida [3]. Mucormycosis is caused by fungi in the order Mucorales of class Zygomycetes. Rhizopus, Mucor, Rhizomucor, Absidia, and Cunninghamella genera species are usually the causative agents in human infections [4]. In the genus Rhizopus, most frequent infectious species is Rhizopus Arrhizus. "Mucormycosis" and “Zygomycosis” have been used interchangeably in medical literature as the majority of human disease is caused by fungi of order Mucorales [5–7].

Mucormycosis can be a life-threatening opportunistic fungal infection. The incidence of mucormycosis among solid organ transplant recipients is 0.4-16 % depending upon the organ being transplanted, and it is 0.2% – 1.2% in renal transplant recipients [8, 9]. In one study, Zygomycetes were the predominant causative agents of nonaspergillus opportunistic fungal infections in solid organ transplant recipients [10]. Usual presentation is within 3–6 months of transplant but may occur many years after the transplant [11]. As in our case, early posttransplant mucormycosis has also been reported [12–14]. Risk factors for mucormycosis infection include immunosuppression, hematological malignancies, diabetes mellitus, steroid use, neutropenia, trauma and burns [15]. Other risk factors are metabolic acidosis, disruption of gastrointestinal mucosal barrier by peptic acid disease, iron overload and deferoxamine treatment [16, 17]. Mucormycosis commonly presents as a sinus-rhino-cerebral disease in solid organ transplant patients [4]. Exposure usually occurs through inhalation, ingestion or inoculation of spores. Other presentations include pulmonary, cutaneous, gastrointestinal, graft organ and disseminated disease. Gastrointestinal mucormycosis is a rare presentation of the disease and is usually due to ingestion of spores. In GI mucormycosis stomach is the most common site of involvement followed by the colon and small bowel. Liver, spleen, and pancreas may rarely be involved as well. Symptoms can be nonspecific and patients can present with abdominal pain, anorexia, abdominal distension, nausea, vomiting and swallowing difficulty. Gastrointestinal bleeding and/or catastrophic gastric or bowel perforation may also be the presenting symptoms. Mucor hyphae have a tendency to grow rapidly and lead to disseminated infection [4]. By invading blood vessels, the fungus can cause thrombosis, tissue necrosis with high morbidity and mortality. Diagnosis is often delayed due to nonspecific presentation and rarity of the disease, therefore a high index of suspicion is required. Beta D glucan testing is not reliable for diagnosing mucormycosis. Diagnosis is usually made after histopathological examination and culture of endoscopy specimens. Rhizopus species are the most common cause of culture-confirmed mucormycosis and histologically, broad, thin-walled, aseptate or sparsely septate hyphae are seen. EGD in gastric mucormycosis shows a discolored mucosa with a shaggy appearance or ulcers with necrotic centers.

Mucormycosis involving the stomach in a renal transplant patient has been reported before [18–22]. Alfano et al. described a case of a 42-year-old female who had a posttransplant upper GI bleed due to gastric ulcers which grew Rhizopus. The patient had a successful outcome with immunosuppression reduction, Amphotericin B and Posaconazole combination. Winkler et al. presented a similar case of a 37-year-old female who developed upper GI bleed on postoperative day 25 due to Rhizopus infection related gastric ulcer that was successfully managed with Amphotericin B. Tinmouth et al. describe a fatal case of gastrointestinal mucormycosis involving the stomach and colon that did not respond to treatment with Amphotericin B, surgical debridement and cessation of all immunosuppression. Radha et al. also describe a fatal case of gastric mucormycosis that did not respond to reduction of immunosuppression and surgical debridement. Kim et al. report a patient with perforating gastric mucormycosis who was successfully managed with emergent total gastrectomy, Amphotericin B and Posaconazole. In contrast, our case was an elderly male who developed esophageal lesions along with gastric ulcer, slightly earlier than as reported in these cases and was successfully treated with Isavuconazole monotherapy and immunosuppressant reduction. Early treatment with antifungal agents and reduction of immunosuppression in solid organ transplant patients is the standard of care. The total duration of antifungal therapy is unclear as there are no definitive guidelines. Treatment duration should be individualized on a case by case basis according to the clinical response.

Despite antifungal therapy, surgical debridement/debulking and reduction of immunosuppressive therapy, mortality can be high due to the aggressive nature of this fungus. The most commonly used antifungal agent for mucormycosis treatment is Amphotericin B [23, 24]. Liposomal Amphotericin B is preferred due to comparative less nephrotoxicity. Posaconazole is also used but its absorption can be erratic and unpredictable and can be associated with severe gastrointestinal side effects such as nausea, vomiting, and diarrhea. Fluconazole, Itraconazole, Voriconazole are not effective. Due to the known nephrotoxic potential of Amphotericin B and drug interaction profiles of other azole compounds with calcineurin inhibitors, Isavuconazole was chosen for antifungal therapy in our case. Isavuconazole is a novel second-generation triazole with a broad spectrum of antifungal activity, approved by the FDA for the treatment of adults with invasive Aspergillosis and mucormycosis. It is available in both intravenous and oral formulations. Since its approval in 2015 [25], Isavuconazole has displayed similar efficacy and a favorable pharmacokinetic profile that has led to less therapeutic drug monitoring, an improved safety profile and less drug-drug interactions [26]. Its once-daily dose oral formulation was favorable in our patient given his extensive medication profile and duration of treatment. Surgical options were not considered in our case as our patient showed a good and prompt response to medical therapy alone.

To the best of our knowledge, this is the first reported case of a patient with gastric mucormycosis after renal transplant successfully treated with Isavuconazole monotherapy. There has been one previous case report published where there was a successful result using Isavuconazole in addition to Amphotericin B and surgical debridement in disseminated pulmonary infection in a renal transplant patient [27]. We acknowledge that we present evidence from a single patient but we highlight the efficacy of Isavuconazole monotherapy in the treatment of mucormycosis in an immunocompromised patient. We also want to highlight the safety profile, low nephrotoxicity and favorable drug interaction profile with calcineurin inhibitors in solid organ transplant patients. Updated guidelines on mucormycosis treatment are needed to reflect the current evidence and to further elucidate the efficacy of Isavuconazole in the treatment of mucormycosis.

## 4. Conclusion

We report a rare case of gastric mucormycosis successfully treated with Isavuconazole monotherapy in a renal transplant patient. Mucormycosis, though rare, can affect gastrointestinal tract in immunocompromised solid organ transplant patients. Symptoms are nonspecific; therefore a high index of suspicion is required to make the diagnosis by endoscopy. Timely introduction of antifungal therapy and the reduction of immunosuppression is the standard of care.
